# Management of a complex traumatic dental injury: Crown, crown‐root, and root fracture

**DOI:** 10.1002/ccr3.3191

**Published:** 2020-08-03

**Authors:** Parisa Sanaei‐rad, Neda Hajihassani, Davoud Jamshidi

**Affiliations:** ^1^ Department of Endodontics School of Dentistry Qazvin University of Medical Sciences Qazvin Iran

**Keywords:** complex, crown fracture, root fracture, trauma

## Abstract

Dental trauma can result in different kinds of injuries based on the extent, direction, and location of the impact. Multidisciplinary management of traumatized teeth is critical for successful treatment and improvement of the prognosis.

## Graphical abstract

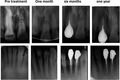

## 1 INTRODUCTION

This paper reports on the management of a complex traumatic injury involving crown, crown‐root, and root fractures, using a multidisciplinary approach, including endodontic, periodontal, orthodontic, and prosthodontic considerations. The healing pattern of the horizontal root fracture involved calcified tissue and the treatment resulted in secured periodontal health and good esthetics.

Traumatic dental injuries have increased during the last few years; such injuries might be caused by falls and collisions, physical activities, and accidents. All over the world, according to many published works, 15.5% of adolescents and young adults aged 7 and 20 years old sustain traumas in at least one permanent tooth.^1^ The anterior teeth, especially the maxillary central incisors, and less frequently, mandibular central incisors, and maxillary lateral incisors comprise the teeth with significant traumas.^2^ Dental traumas can result in different kinds of injuries depending on the extent, direction, location of the impact, and tooth development stage. According to current clinical classifications, crown, crown‐root, and root fractures are the common injuries to the hard dental tissues and the pulp.^3^ Crown fractures involve fractures or cracks of the enamel and/or dentin, with or without loss of tooth substance. They are defined as complicated, in the case of pulp exposure, or uncomplicated when the pulp is not exposed after trauma. When a fracture involves enamel, dentin, and cementum and extends below the gingival margin, it is defined as crown‐root fracture. In root fractures, only the root structure including dentin, cementum, and pulp is involved and it can be localized at the apical, middle, or cervical third.^3^

Following diagnosis of a traumatic injury, a treatment plan should be established according to the type of the fracture, stage of tooth development, endodontic prognosis and periodontal, restorative and prosthodontics considerations. For complicated crown fractures, treatment options are vital pulp therapy and pulpectomy. These options can be considered for crown‐root fractures; however, the prosthetic coronal restoration is unfavorable because the fracture line extends to the root below the crestal bone. Three approaches can be selected to reestablish the biological width and make the tooth restorable: (a) orthodontic extrusion, (b) surgical extrusion, and (c) crown lengthening. Orthodontic extrusion is superior due to the better management of esthetic concerns and coronal displacement of crestal bone and gingival margin. To avoid any relapse, circumferential supracrestal fiberotomy should be performed after extrusion. For root fractures, repositioning the coronal fragment and immobilizing the tooth with splint are choices of treatment. When the fracture is located in the apical or middle third of the root, the prognosis is favorable.^4^, ^5^, ^6^

This case report describes a complex traumatic dental injury involving crown, crown‐root, and root fractures which were endodontically managed using a multidisciplinary approach including periodontal, orthodontic, and prosthodontics considerations.

## 2 CASE REPORT

**FIGURE 1:**
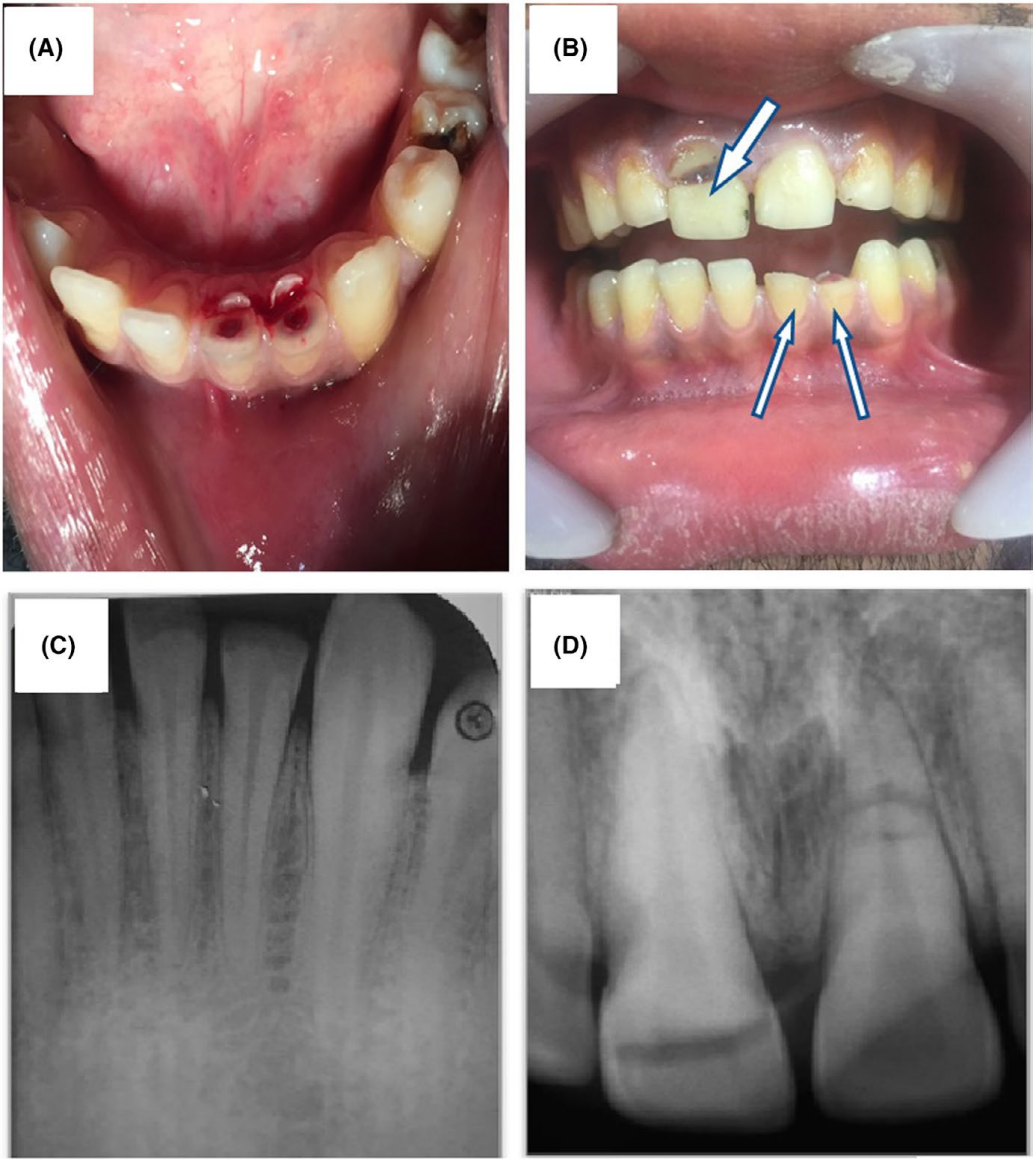
Pre‐operative intra oral pictures (A, B) and periapical radiographs (C, D). The arrows show traumatized teeth

This report involves a 22‐year‐old male patient reporting to the Department of Endodontics, Qazvin University of Medical Sciences, with the chief complaint of fractured teeth caused by trauma with a wrench, 3 days before. The medical history was normal and classified as ASA I. In clinical evaluations, extra oral examination showed no bleeding, fracture of facial bones, or draining sinuses. Intra oral examination showed no swelling and sinus tract; teeth #8, #23, and #24 were traumatized with pulp exposure (Figure 1A,B). According to the pulp sensibility test, teeth #23 and #24 showed a severe response but tooth #8 exhibited a mild response. None of the teeth above were tender to percussion/palpation. Interestingly, tooth #9 did not show any response to cold/heat test, exhibited tenderness to periapical tests and showed grade I mobility. The crown fractures of traumatized teeth were revealed on the panoramic radiograph. Intra oral periapical radiograph demonstrated a horizontal root fracture in the apical third of tooth #9; crown and oblique crown fracture line extending subgingivally in tooth #8 (Figure 1C,D). The radiographic examination also revealed crown fractures of teeth #23 and #24 and no damage to the adjacent teeth. Based on the clinical and radiographic findings, the patient was diagnosed with complex traumatic dental injuries involving crown‐root fracture and root fracture in teeth #8 and #9, respectively. Teeth #23 and #24 were also diagnosed with complicated crown fracture.

**FIGURE 2:**
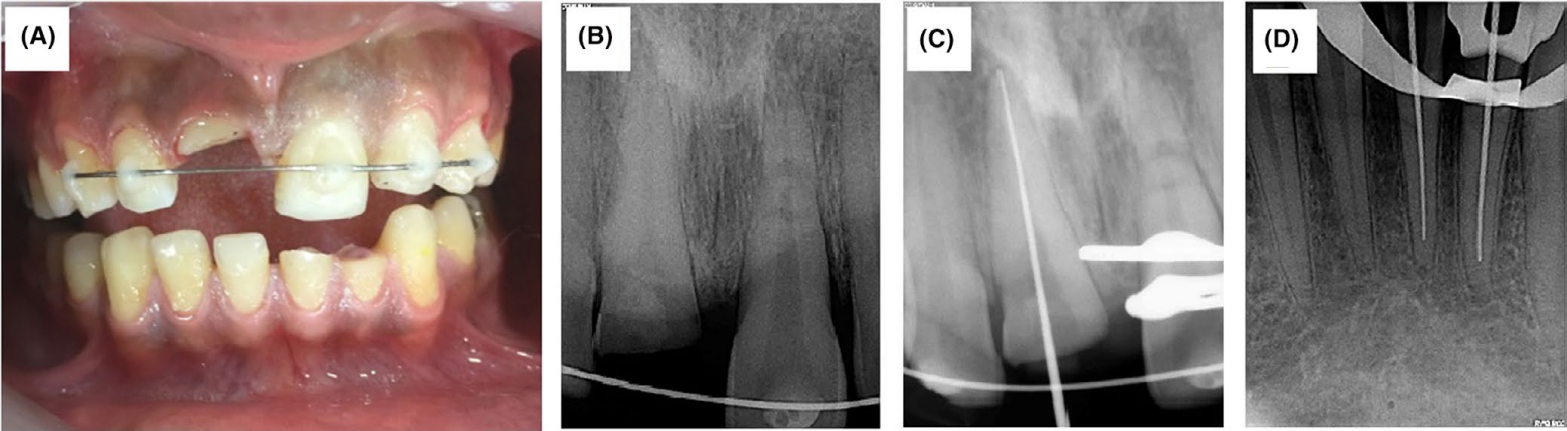
Splinting of tooth #9 (A, B) and working length determination of tooth #8 (C), and teeth #23, #24 (D)

**FIGURE 3:**
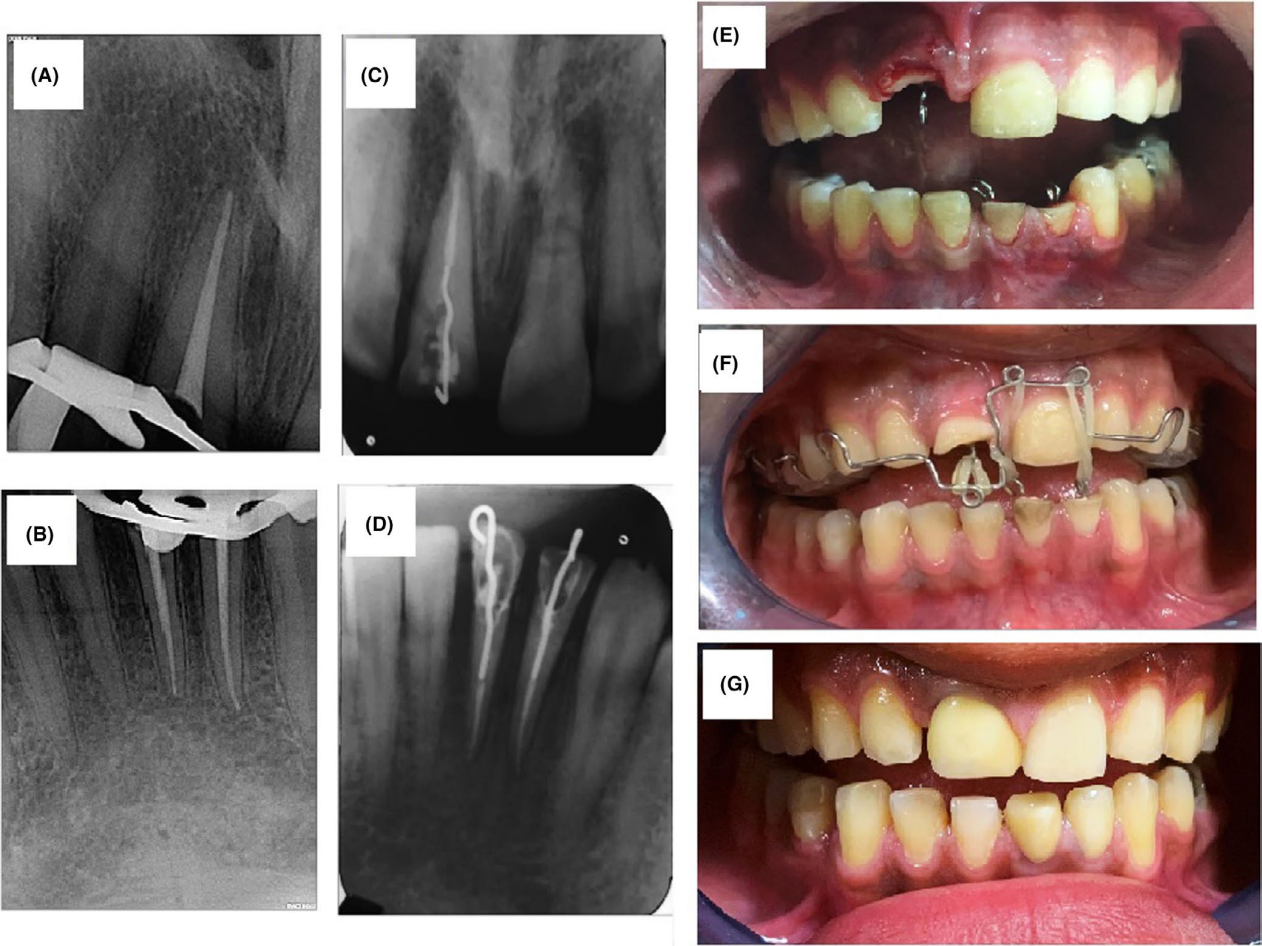
Postoperative radiograph of root canal treatment of tooth #8 (A) and teeth #23, #24 (B), Radiograph of hook cementation inside root canals of tooth #8 (C) and teeth #23, #24 (D), clinical images before (E) and after (F) application of orthodontic appliance and elastics and cemented post and core in 6‐mo follow‐up (G)

**FIGURE 4:**
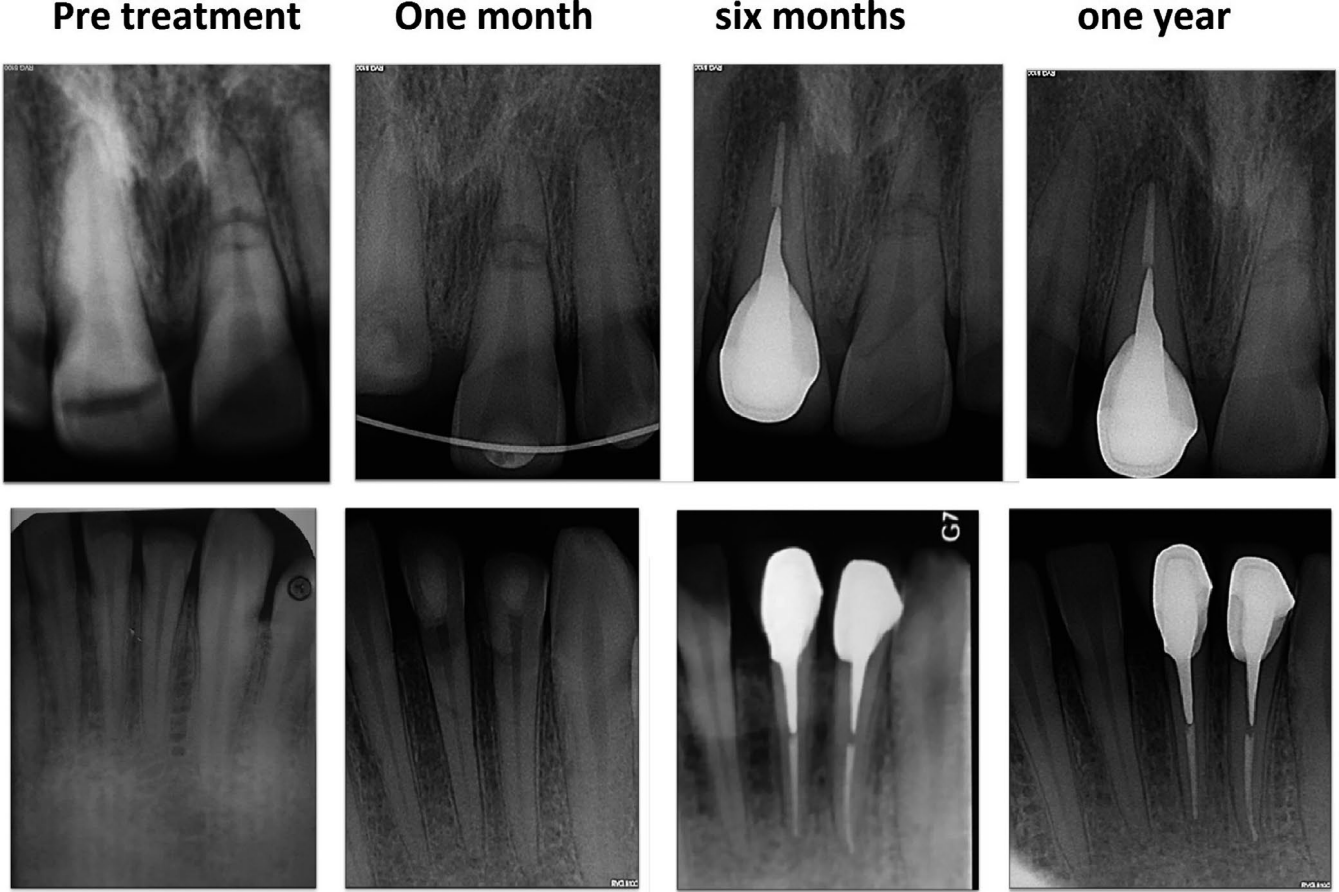
Follow‐up radiographs for teeth #8 and #9 (top) and teeth #23 and #24 (bottom)

All the traumatized teeth were subjected to emergency treatment on the first visit. After obtaining informed consent, to evaluate the fractures and determine the restorability, the lingual mobile segment of teeth #8 was removed (this was a part of the diagnosis). To immobilize tooth #9, semi‐rigid splinting (33) was performed using 0.5 mm diameter round stainless steel wire and composite resin (Figure 2A,B). Also, emergency pulpectomy was initiated for teeth #8, #23, and #24. After local anesthesia (2% lidocaine with 1:80 000 epinephrine; Daroupakhsh) and rubber dam isolation, a fissure diamond bur in a high‐speed handpiece with water spray was used to prepare an access cavity. The working length was determined by Root ZX electronic apex locator (J Morita MFQ) and confirmed with x‐ray (Figure 2C,D). Cleaning and shaping of the root canal system were completed using the crown‐down technique followed by irrigation with normal saline solution and 5.25% sodium hypochlorite. The root canals were dried with sterile paper points. A creamy mix of calcium hydroxide as the intracanal dressing was placed with a lentulo spiral. Finally, the access cavity was sealed temporarily with reinforced Zinc Oxide‐Eugenol. The patient was recalled 1 month later. In the next visit, after splint removal, obturation was performed with gutta‐percha and AH26 sealer by the lateral compaction technique (Figure 3A,B). In the 8‐week follow‐up visit, the existing gutta‐percha was removed to within 5 mm of the root apex with a selection of Gates‐Glidden drills in teeth #8, #23, and #24. A temporary hook was cemented inside the canals using zinc phosphate cement to aid in extrusion (Figure 3C,D,E). A customized orthodontic appliance was made for the patient and orthodontic elastic (size 1/8 for teeth# 23, and #24, and size 3/16 for tooth #8) was applied from the hook to the specially designed loop for rapid extrusion (Figure 3F). One month later, circumferential supracrestal fiberotomy was performed to avoid any relapse. After the active period of extrusion, the teeth were stabilized for 8 weeks, using a splint. Following the retentive phase, the retainer was removed and the prosthodontic treatment planning and a primary impression were undertaken (Figure 3G). A custom‐made post and core was prepared using a direct technique followed by permanent cementation. In the 6‐month follow‐up, the teeth were asymptomatic with no sign of root resorption or lesion (Figure 4). In the 1‐year follow‐up, tooth #9 was responsive to pulp sensibility tests. The previously treated teeth did not show any pain, sign, symptoms, and tenderness to percussion. Interestingly, the healing pattern of the horizontal root fracture was demonstrated to be with calcified tissue.

## 3 DISCUSSION

This case report presents a multidisciplinary approach for the management of a complex traumatic dental injury involving crown, crown‐root, and root fractures. Root fractures are relatively rare among dental traumas comprising <3% of all dental injuries.^7^ In horizontal root fractures, pulp necrosis might develop in the case of coronal segment displacement; therefore, root canal treatment is required.^8^ The splinting period for teeth with horizontal root fracture depends on the location of the fracture. If the fracture is located in the apical or middle third of the root, a 4‐week splinting period is recommended and when the fracture is near the cervical area of the tooth, it is suggested that splinting continues for a longer period. The healing of root fracture is affected by three factors: (a) the degree of dislocation and mobility, (b) maturity, and (c) the quality of development.^9^, ^10^ After healing, three types of tissues can be described based on examinations: (a) calcified tissue (b) interproximal connective tissue, and (c) interproximal bone and connective tissue. Healing might not be confirmed due to the formation of interproximal inflammatory tissue.^11^ In the present case, in a 1‐year follow‐up, the fracture line of tooth #9 was visible in the radiograph but the fragments were in close contact, indicating that healing involved a calcified tissue. The successful outcome of treatment was also confirmed while the tooth was shown to be asymptomatic and responsive to pulp sensibility tests with no response in the emergency therapy session. According to the current classification, the fracture line is below the gingival margin in crown‐root fractures in contrast to crown fractures. In teeth #23 and 24 with crown fracture and tooth #8 with crown‐root fracture, the pulp was involved; therefore, these fractures were termed “complicated.” If the exposed pulp is left untreated, necrosis will occur and the prognosis of treatment outcome depends on the type and time sequence of pulp necrosis. The choice of treatment, pulpectomy, or vital pulp therapy is also influenced by factors such as tooth maturity, the time between trauma and treatment and restorative plan. In the present case, the tooth was mature, more than 48 hours had elapsed since trauma, and the restorative plan was a post and core. Due to the favorable prognosis, pulpectomy was selected as the treatment of choice for teeth #8, 23, and 24. In the case of tooth fracture and deep carious lesions, clinical crown lengthening is performed to expose sound tooth structure which improves restoration retention, appropriate placement of restoration margins and patient esthetics. It can be performed using the following three procedures: (a) orthodontic extrusion, (b) surgical extrusion, and (c) crown lengthening. The selection of an appropriate technique is affected by several factors such as esthetics, crown‐to‐root ratio, root morphology and proximity, tooth position, and restorative treatment plan.^12^, ^13^ In crown lengthening, apical shifting of the gingival margin might interfere with the patient esthetic. It is also less conservative and might lead to sacrificing supporting bone. In this case, orthodontic rapid extrusion was performed to achieve sufficient tooth structure for further permanent restoration. In rapid extrusion, coronal shift of marginal bone is slight and periodontium movement is avoided with the fiber tension. Therefore, circumferential supracrestal fiberotomy must be performed to avoid any relapse.^14^, ^15^ In addition, before final restoration, the teeth were retained in their new position for 8 weeks for bone remodeling and reorganization of periodontal fibers to prevent relapse. In the present case, a complex dental trauma including crown, crown‐root and root fractures was managed by a multidisciplinary approach with periodontal, orthodontic and prosthodontic considerations. The outcome of the management was favorable. However, the use of orthodontic appliances and a long treatment period can be mentioned as the major drawbacks of this approach. Before treatment, the patient was informed about different treatment options, and after treatment planning, the patient was requested to be fully committed and have a proper compliance.

## CONFLICT OF INTEREST

The authors have declared that no conflict of interest exists.

## AUTHOR CONTRIBUTIONS

PS: reviewed the literature, developed the concept and design of the study, performed the procedure, and drafted the manuscript. NH: reviewed the literature and was involved in data analysis/interpretation and drafting the manuscript. DJ: involved in concept/design, analysis and data collection and drafting the manuscript.

## ETHICAL APPROVAL

This case report meets the ethical guidelines and adheres to Iran's local legal requirements.
